# Mixed response on regorafenib treatment for GIST (gastro-intestinal stromal tumor) according to ^18^F–FDG-PET/CT

**DOI:** 10.1186/s12885-018-4154-7

**Published:** 2018-03-05

**Authors:** Donatienne Van Weehaeghe, Olivier Gheysens, Vincent Vandecaveye, Patrick Schöffski, Koen Van Laere, Christophe M. Deroose

**Affiliations:** 1 Nuclear Medicine, University Hospitals Leuven, Herestraat 49, 3000 Leuven, Belgium; 2 Radiology, University Hospitals Leuven, Herestraat 49, 3000 Leuven, Belgium; 3 Department of oncology, University Hospitals Leuven, Herestraat 49, 3000 Leuven, Belgium

**Keywords:** GIST, ^18^F–FDG-PET/CT, Regorafenib, Follow-up, Attenuation-artefact, Respiratory motion, Case report

## Abstract

**Background:**

Gastro-intestinal stromal tumors (GISTs) are very rare tumors of the gastro-intestinal tract, originating from the interstitial cells of Cajal or a common cell precursor which both express type III tyrosine kinase receptors. Regorafenib is an oral multi-kinase inhibitor used to treat gastro-intestinal stromal tumors. To our knowledge this is the first case in literature to show the response of regorafenib on PET.

**Case presentation:**

A 37-year-old male with lower abdominal pain and weight loss was referred to our hospital. Abdominal ultrasound and computed tomography (CT) showed diffuse peritoneal implants. Surgical specimen histology showed a GIST with c-KIT exon 11 deletion (c.1708_1728del) and treatment with imatinib 400 mg/day was initiated. Due to disease progression illustrated on baseline versus follow-up ^18^F–FDG-PET/CT scans therapy was switched to imatinib 800 mg/day and later to sunitinib 50 mg/day. Upon further disease progression 10 months later, third line treatment with regorafenib 160 mg/day was initiated. ^18^F–FDG-PET/CT showed the metabolic responses after 4 months regorafenib treatment ranging from complete response to the appearance of a new lesion in the liver. The new hypermetabolic lesion was only seen on the non-attenuation-corrected images because of breathing motion artifact.

**Conclusion:**

This case illustrates that metabolic response can occur in GIST lesions without morphological response after third line regorafinib treatment. Furthermore this is the first case in literature to show regorafinib response on PET.

## Background

Gastro-intestinal stromal tumors (GISTs) are the most common mesenchymal tumors of the gastro-intestinal tract. However they are very rare, accounting for about 1% of the tumors of the gastro-intestinal tract. These tumors originate from the interstitial cells of Cajal or other common cell precursors which express tyrosine kinase receptors (type III). They are sometimes called the pacemaker cells of the gut. Treatment consists of surgical resection with or without adjuvant/neo-adjuvant therapy with an oral multi-kinase inhibitor like regorafinib [1–3].

Regorafenib is an oral multi-kinase inhibitor used to treat metastatic GISTs after progression on standard treatment. It significantly improve progression-free survival compared with placebo in patients [3]. To our knowledge this is the first case in literature to show the response of regorafenib on ^18^F–FDG PET/CT.

## Case presentation

A 37-year-old male complaining about lower abdominal pain and weight loss was referred to our hospital. He reported a weight loss of 5 kg in the last 3 months. An abdominal ultrasound and computed tomography (CT) were performed as work-up. Both examinations showed diffuse peritoneal implants.

## Discussion

**Fig. 1:**
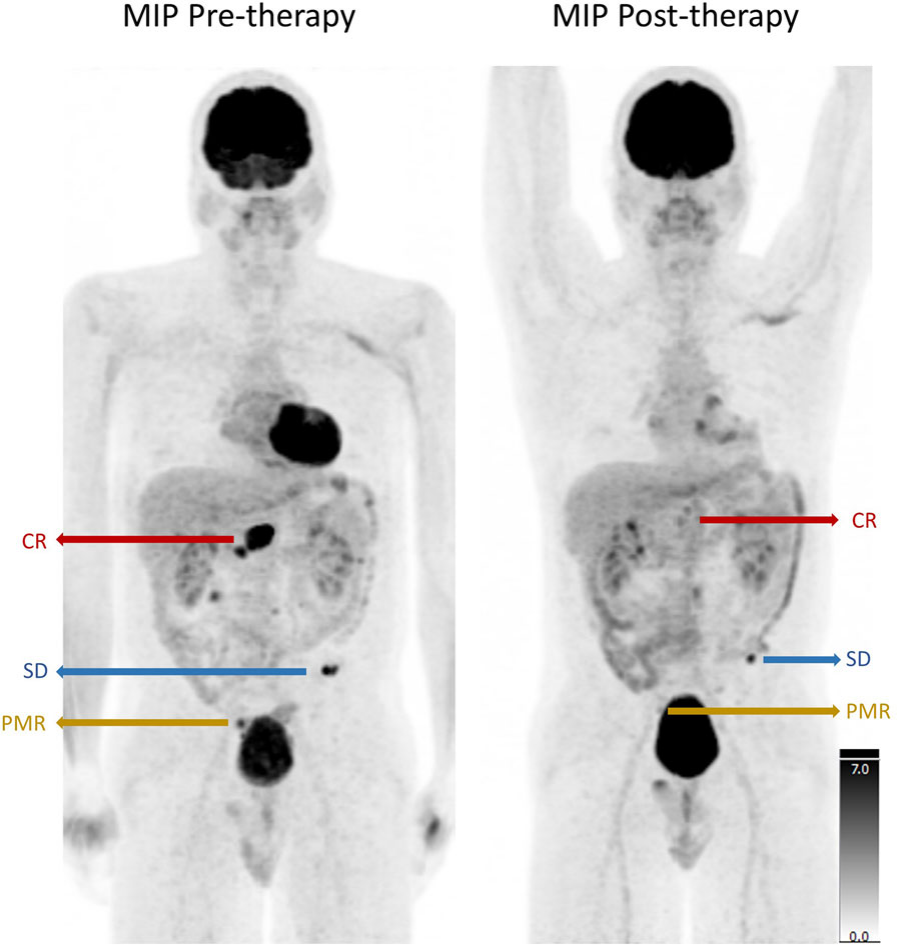
Maximal intensity projection (MIP) images of the ^18^F–FDG-PET scan at baseline and after regorafenib treatment. CR = complete response; SD = stable disease; Deep PMR = deep partial metabolic response

Surgical exploration and debulking was performed to obtain a tumor specimen for histopathological examination. Histological examination of this specimen showed a GIST with c-KIT exon 11 deletion (c.1708_1728del). As ^18^F–FDG PET has been shown of significant value in evaluating treatment response in GISTs, high dose contrast-enhanced ^18^F–FDG PET/CT scans (374.9 ± 17.2 MBq; approximately 60 min after tracer injection) were performed both before treatment and after every therapy switch to evaluate treatment response [4]. ^18^FDG-PET/CT performed for tumor staging showed multiple tumor localizations in the small bowel, the sigmoid and mesenterium without signs of extra-abdominal disease. Treatment with imatinib 400 mg daily was started with follow-up ^18^F–FDG PET/CT 2 months later showing disease progression. The dose was increased to 800 mg daily but follow-up ^18^F–FDG PET/CT 3 months later again revealed disease progression. A switch to sunitinib 50 mg once a day was performed. Upon further disease progression on the ^18^F–FDG PET/CT 10 months later, third line treatment with regorafenib 160 mg/day was initiated with a mixed response on ^18^F–FDG-PET/CT 4 months after treatment initiation with regorafinib (Fig. 1). There was one lesion with a complete metabolic response (CR), one with a partial metabolic response (PMR) and one with stable disease (SD) according to the EORTC criteria for ^18^F–FDG-PET response [5].

**Fig. 2:**
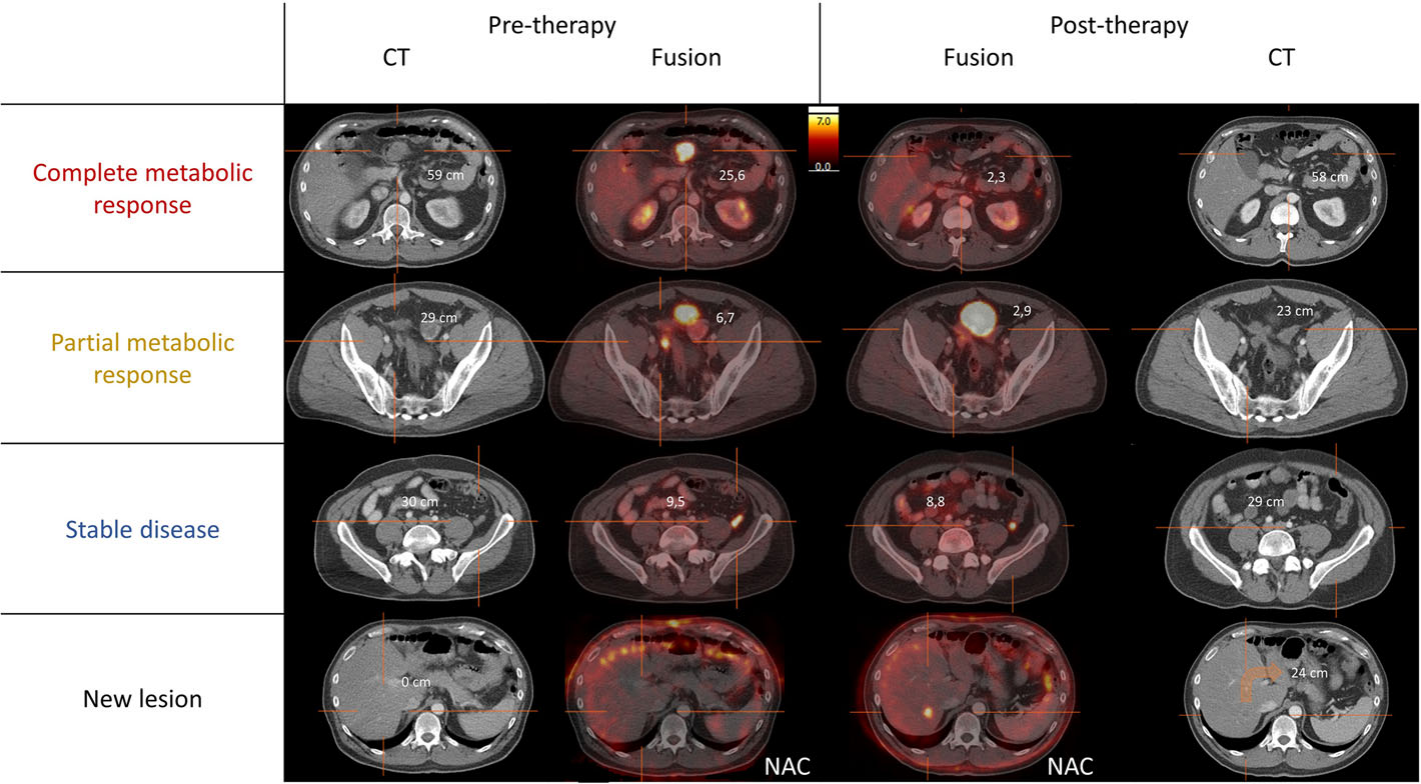
Pre- and post-therapy fused PET/CT and CT images of the different lesions. NAC = non-attenuation-corrected images

**Fig. 3:**
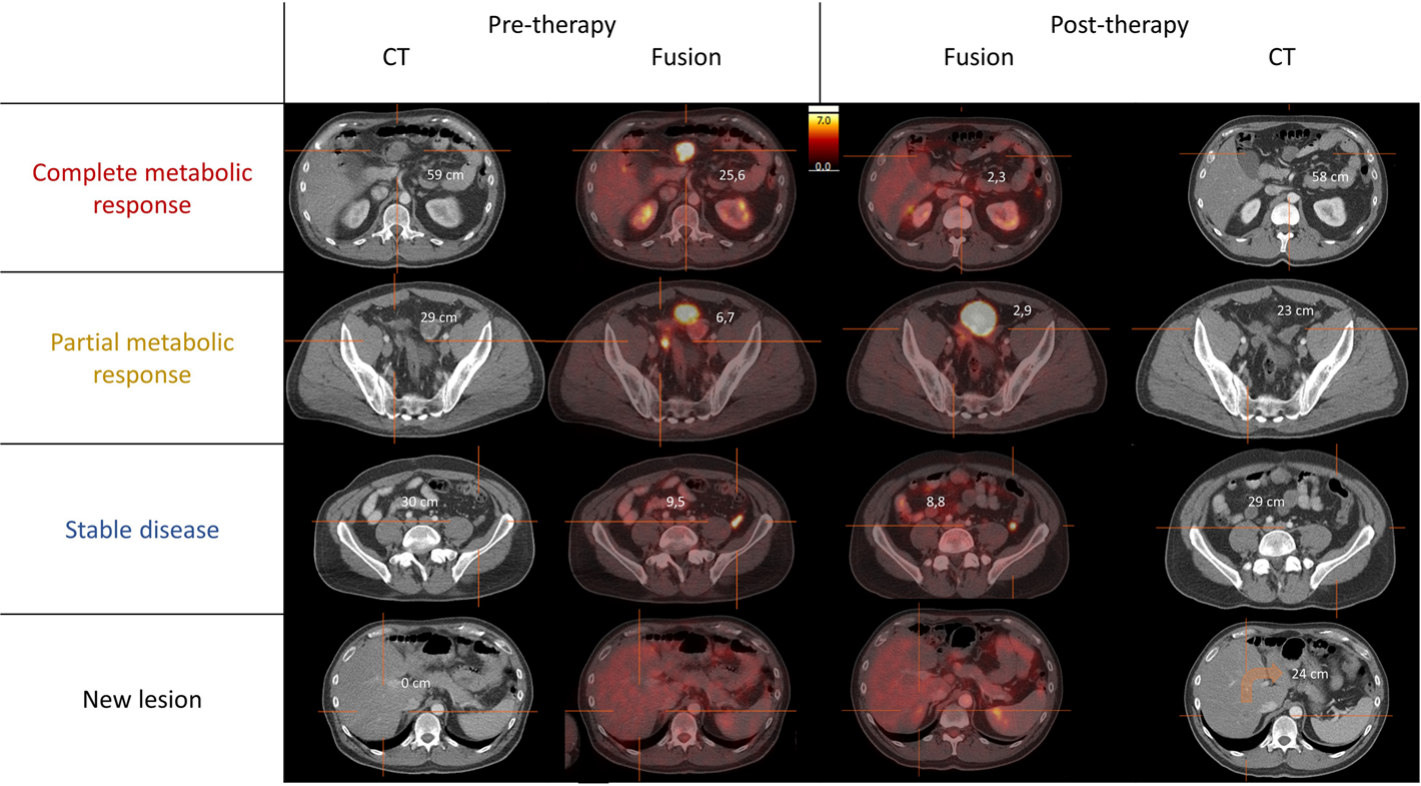
Pre- and post-therapy fused PET/CT and CT images of the different lesions

Pre- and post-therapy with regorafinib fused PET/CT and CT images with the differences in maximal standardized uptake value (ΔSUV_max_) and differences in maximal diameter (Δdiam_max_) are shown in Figs. 2 and 3. The lesion with complete metabolic response had a ΔSUV_max_ of − 91% and a Δdiam_max_ of − 1.7%. The lesion with the partial metabolic response had a ΔSUV_max_ of − 56% and a Δdiam_max_ of − 21%. Both lesions were stable disease on CT scan according to the RECIST1.1 criteria. [6]. The lesion with stable disease on PET had a ΔSUV_max_ of − 8.0% and a Δdiam_max_ of − 3.3%. The total volume of the lesion with complete metabolic response was 19.9 cm^3^ pretherapy and 17.6 cm^3^ posttherapy. The lesions with partial metabolic response and stable disease did not change in volume and were respectively 4.1 cm^3^ and 3.6 cm^3^. The volume of the new lesion was 3.2 cm^3^. No histological confirmation of this new lesion was obtained due to the general condition of the patient. However, this lesion increased both in volume and metabolism on follow-up scans, compatible with a true positive new tumoral lesion.

Besides these previously known lesions, a new hypermetabolic lesion was seen on the non-attenuation-corrected (NAC) ^18^F–FDG-PET images. It corresponded to a new hypodense liver lesion on CT, implying a new liver metastasis. However, the lesion was not visible on the attenuation-corrected (AC) and MIP images because of breathing motion-induced misregistration and subsequent lung density attenuation correction, which strongly reduces the apparent uptake in the lesion. This lesion has important consequences for the patient with regard to further treatment options (switch to another tyrosine kinase inhibitor) and illustrates the importance to look at NAC images on all oncological scans, in particular not to miss liver lesions within the liver dome [7, 8].

## Conclusion

This case illustrates that metabolic response to third line regorafinib treatment can occur in GIST lesions without morphological response. Therefore, even though it did not affect treatment decision in this case, this finding highlights the importance of ^18^F–FDG PET scans in the evaluation of treatment response in future GIST tumors cases.

## Abbreviations

18F-FDG PET: [18]Fluor-Fluorodeoxyglucose Positron Emission Tomography
AC: Attenuation-corrected
c.1708_1728del: c-KIT exon 11 deletion
CR: Complete metabolic response
CT: Computed tomography
GISTs: Gastro-intestinal stromal tumors
MIP: Maximal intensity projection
NAC: Non-attenuation-corrected
PMR: Partial metabolic response
RECIST: Respons Evaluation Criteria in Solid Tumours
SD: Stable disease
Δdiam_max_: Maximal diameter
ΔSUV_max_: Maximal standardized uptake value
